# Overweight and obese adults have low intentions of seeking weight-related care: a cross-sectional survey

**DOI:** 10.1186/1471-2458-14-582

**Published:** 2014-06-11

**Authors:** Jacqueline Tol, Ilse C Swinkels, Dinny H De Bakker, Cindy Veenhof, Jaap C Seidell

**Affiliations:** 1 NIVEL (The Netherlands Institute for Health Services Research), P.O. box 1568, Utrecht 3500 BN, The Netherlands; 2 TRANZO (Tilburg University, Scientific Centre for Transformation in Care and Welfare), P.O. box 90153, Tilburg 5000 LE, The Netherlands; 3 VU University of Amsterdam, De Boelelaan 1085, Amsterdam 1081 HV, The Netherlands

**Keywords:** Overweight, Obesity, Weight change, Patient’s acceptance of health care, Perception, Dietary services

## Abstract

**Background:**

The prevalence of obesity is growing worldwide. Obesity guidelines recommend increasing the level of weight-related care for persons with elevated levels of weight-related health risk (WRHR). However, there seems to be a discrepancy between need for and use of weight-related care. The primary aim of this study is to examine predisposing factors that may influence readiness to lose weight and intention to use weight-related care in an overweight population.

**Methods:**

A population-based, cross-sectional survey was conducted. Data were collected using an online self-administered questionnaire sent to a population-representative sample of 1,500 Dutch adults on the Health Care Consumer Panel (n = 861 responded). Data were used from individuals (n = 445) with a mildly, moderately or severely elevated level of WRHR. WRHR status was based on self-reported data on Body Mass Index, risk assessment for diabetes mellitus type 2 (DM2) and cardiovascular disease (CVD), or co-morbidities.

**Results:**

55.1% of persons with increased WRHR were ready to lose weight (n = 245). Depending on level of WRHR; educational level, marital status, individuals with an accurate perception of their weight and better perceptions and expectations of dietitians were significantly related to readiness to lose weight. Most of them preferred individual weight-loss methods (82.0% of n = 245). 11% (n = 26 of n = 245) intended to use weight-related care. Weight-related care seeking was higher for those with moderate or severe WRHR. Expectations and trust in dietitians did not seem to influence care seeking.

**Conclusions:**

Many Dutch adults who are medically in need of weight-related care are ready to lose weight. Most intend to lose weight individually, and only a few intend to use weight-related care. Therefore, obesity prevention initiatives should focus on monitoring weight change and weight-loss plans, and timely referral to obesity management. However, many people are not ready to lose weight. For this group, strategies for behaviour change may depend on WRHR, perceptions of weight and dietitians, educational level and marital status. Obesity prevention initiatives should focus on increasing the awareness of the seriousness of their condition and offering individually appropriate weight management programmes.

## Background

**Figure 1:**
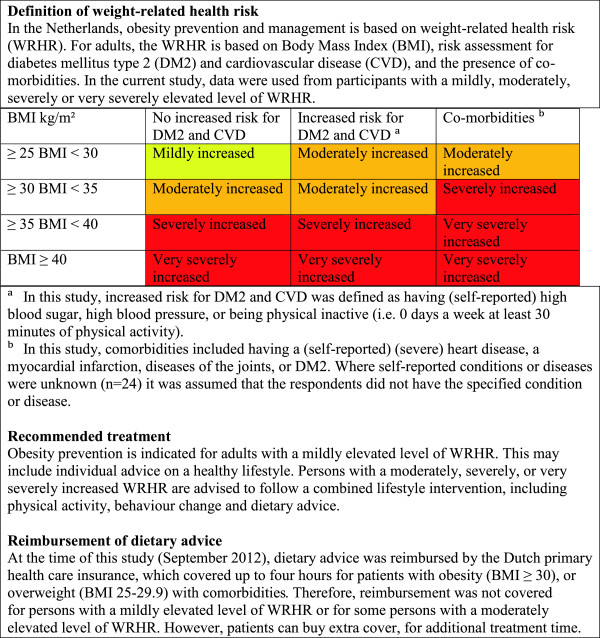
Contents of obesity prevention and management by level of weight-related health risk.

Obesity prevention and the effective management of those with obesity constitute a public health challenge. Worldwide, the prevalence of obesity has increased in recent decades [1]. In the Netherlands, the prevalence of obesity among adults has risen from 5% in 1981 to 12% in 2011 and the prevalence of overweight from 28% to 36% [2]. The increase is considered to be the result of a combination of environmental, biological and social factors [3]. Because of the complexity of this multi-factorial problem, many people need help with the prevention of weight gain and with weight-management. The rationale for adult weight management in Dutch primary healthcare is based on the health risks associated with overweight and obesity. In general, Dutch obesity guidelines recommend increasing the level of weight-related care for persons with elevated levels of weight-related health risk (WRHR) (see Figure 1) [4]. Dietary treatment is an important aspect of weight management, which can be given by a multidisciplinary team of healthcare professionals, including dietitians. Dietetic treatment has been demonstrated to be a moderately effective weight loss strategy for overweight persons in primary health care [5].

In general, weight-related care use may depend on several aspects including medical need, enabling factors (such as insurance and accessibility) and predisposing factors (such as demographics and health beliefs) [6,7]. The medical need for weight-related care is high due to the prevalence of overweight and obesity. In the Netherlands, weight-related care use will likely also be influenced, albeit to a lesser extent, by enabling factors, since dietary treatment is partly reimbursed by health insurers (see Figure 1). Moreover, direct access (self-referral) to dietitians is available. In spite of this, the actual use of dietetic care services is relatively low: approximately 2% of the Dutch population used dietetic healthcare for various reasons in 2010 [8]. This raises questions about the type of individuals who are ready to lose weight and use weight-related care, as well as the influencing factors.

In analysing individuals’ readiness to lose weight, the trans theoretical model of change suggests how change occurs. According to this model, behaviour change occurs over time and involves different stages: precontemplation, contemplation, preparation, action, maintenance and termination. Persons in the first two stages of change are ambivalent about making change. They may benefit from counselling about the harm caused by their current behaviour and the benefits of change. Those at the preparation stage, or further, generally have a plan of action. The middle stages of preparation and action are the most volatile, and people are likely to progress or regress, depending on the help they receive [9]. These individuals and those in further stages are likely to make progress and therefore appear to be ready for weight-related care. A number of factors can impact readiness for weight change, including demographics (i.e. gender, race, education) [10,11] or psychological factors (attitudes, beliefs, and intentions) [11-14].

Social-psychological factors that may influence the uptake of weight-related care include beliefs about weight, perceptions and expectations of care providers who give dietary advice, and trust in care providers [7]. Better knowledge about predisposing factors such as perceptions and expectations of, and trust in care providers may contribute to our understanding of the relatively low use of dietary health services. Few studies have been carried out on public perceptions and expectations of care providers who give dietary advice. A study by Crocker showed doctors were the preferred choice for nutritional information, followed by dietitians; however, younger people preferred advice from health food shops [15]. Gorton et al. showed that dietitians perceived themselves to be one of the last resorts for weight loss. However, clients ranked them as the second choice after exercise [16]. The authors report that clients hold a variety of expectations regarding private practice dietitians and that initial perceptions were not particularly favourable.

In sum, little research is available about the types of persons with elevated levels of WRHR who are ready to lose weight, those who are intending to use weight-related care, and those who are not. More knowledge about the influencing factors might contribute to our understanding of health behaviour in an overweight population and improve policies aimed at activating people to reduce WRHR. Therefore, the primary aim of this study was to examine predisposing factors that may influence readiness to lose weight and important reasons for not being ready to lose weight in an overweight population. The secondary aim of the study was to examine predisposing factors that may influence intention to use weight-related care in an overweight population ready to lose weight.

## Methods

### Sample

Data were collected in September 2012 through an online survey, sent out to a sample of 1,500 members of the Dutch Health Care Consumer Panel [17,18]. The sample was drawn from 6000 panel members aged 18 years and older. Stratified random sampling was used in order to obtain a sample of panel members that was representative by age and gender of the Dutch population aged 18 years and older. The panel members have agreed to answer questions about healthcare on a regular basis. General information was available concerning the participants (e.g. age, gender, ethnicity, level of education, net monthly household income in euros, marital status, and self-reported general health status) as these characteristics were documented upon entry to the panel and are updated regularly. Data were processed anonymously. The Dutch Health Care Consumer Panel is registered with the Dutch Data Protection Authority (no. 1262949). The study does not fall within the scope of the Medical Research Involving Human Subjects Act and therefore does not require ethical approval [19].

### Questionnaire

**Figure 2:**
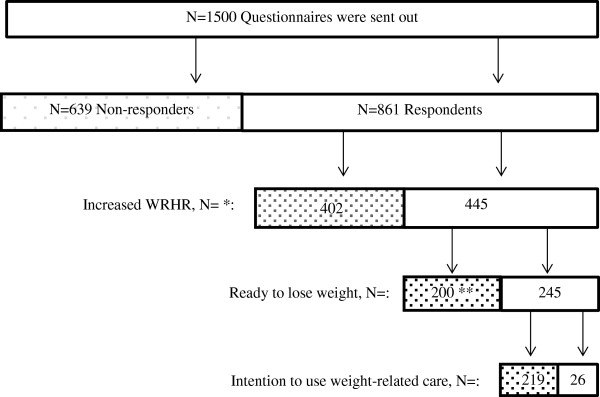
**Flowchart of study participants.** Legend: No , Yes . * Weight-related health risk (WRHR) could not be determined for 14 respondents since they did not enter details of their height and weight. Body mass index (BMI) could therefore not be calculated. Since BMI was missing at random, these 14 respondents were excluded from the analysis. ** 200 respondents with an elevated level of weight-related health risk (BMI ≥ 25) were not ready to lose weight. They were asked to report the three most important reasons for not being ready to lose weight.

For the purpose of this population-based, cross-sectional study, we developed a questionnaire which was filled out by the sample of panel members. Data were used from a subgroup of respondents with a medical need for obesity prevention or management, including persons with mildly, moderately, severely, or very severely increased WRHR (see Figure 2).

The questionnaire contained questions on age, gender, health conditions, symptoms or diseases, body weight, height, level of physical activity, perception of body weight, readiness to lose weight, intention to use weight-related care, past weight-related care use, perceptions of dietary advice from care providers, expectations of dietitians and a rating for trust in dietitians. An additional pdf file shows the questionnaire in more detail (see Additional file 1).

Dichotomous variables were created for level of physical activity (<5 days a week / ≥ 5 days a week), accurate perception of body weight (no/yes), perceiving dietitians as suitable care providers (no/yes) and readiness to lose weight (ready to change/not ready to change). The following question was used regarding level of physical activity: “How many days a week do you exercise for at least 30 minutes per day?” Having an accurate perception of body weight was defined using the question: “To what extent do you agree with the following statement; I believe I am too heavy”. Respondents with a BMI ≥25 who answered “strongly agree” or “somewhat agree” were defined as having an accurate perception. Those who answered “somewhat disagree” or “strongly disagree” were categorised as having an inaccurate perception. Perceiving dietitians as suitable care providers was defined using the question “Please indicate the extent to which you consider the following care providers to be qualified to give dietary advice?” Respondents who answered “very unqualified” or “somewhat unqualified” were defined as not perceiving dietitians as suitable care providers. Those who answered “somewhat qualified” or “very qualified” were categorised as perceiving dietitians as suitable care providers.

Readiness to lose weight was defined using the question, “Do you plan to start losing weight?” Respondents with a BMI ≥25 who answered “Yes, I’m planning to start during the next month” or “Yes, I am currently changing” were defined as ready to change. Those who responded “No” or “Yes, I’m planning to change but not in the short term” were classified as not ready to engage in weight-related behaviour change. Additionally, they were asked about the most important reasons (maximum three out of fifteen) for not planning on losing weight, or at least not in the short term. Respondents who were ready to change were asked about their weight loss plans (multiple choice), including the intention to use weight-related care from a care provider.

Face validity was assessed by the authors of this study and two researchers of the Dutch Health Care Consumer Panel. In addition, the questionnaire was commented on by the programme committee of the Dutch Health Care Consumer Panel (i.e. by the Ministry of Health, Welfare and Sport and the Federation of Patients and Consumer Organizations in the Netherlands) and the Dutch Association of Dietetics. Moreover, the questionnaire was piloted on 10 adults who were not included in this study sample.

### Data-analysis

Univariate and bivariate analyses were performed to examine predisposing factors that may influence readiness to lose weight, and, subsequently, intention to use weight-related care. Results on readiness to lose weight were stratified by WRHR. The small sample size of respondents intended to use weight-related care limited further statistical analysis. Categorical data were tested using Chi-square tests and Fisher exact tests for groupings with < 5 responders in a field, to test for a significant difference in the dichotomous outcome variables of readiness to lose weight and intention to use weight-related care. Furthermore, a scale was developed to test the overall influence of the nine items on the expectations regarding dietitians of respondents ready to lose weight and intending to receive weight-related care. Confirmatory factor analysis was used to evaluate the factor structure. The Kaiser-Meyer-Olkin measure of sampling adequacy was high (0.85) and one factor had an eigenvalue of greater than one (3.78). The data demonstrated strong internal reliability with Cronbach alpha of 0.87. Consequently, average scale scores were calculated for each respondent. Higher scores (range 1–4) indicated better expectations of dietitians. Differences in the expectations scores between readiness to lose weight, and, subsequently intention to use weight-related care were examined using Wilcoxon rank sum tests. In addition, the difference in the trust-rating of dietitians between readiness to lose weight and intention to receive weight-related care was tested using Student’s t-test. A multivariate logistic regression model, stratified by WRHR, was created to examine the impact of each independent variable on readiness to lose weight. Covariates with p < 0.15 in bivariate analysis were selected for inclusion in the logistic model since more traditional levels may fail to identify variables known to be important [20]. Covariates were then removed from the model if they were non-significant (p < 0.05). The model was tested for multi-collinearity. Odds ratios and 95% CIs were calculated. Data were analysed in STATA (Version 12, 2011, STATACorp, College Station Texas).

## Results

### Response

The response rate for this study was 57% (n = 861) (see Figure 2). Respondents were significantly older compared with non-responders (mean age 54.5 ± 14.6 versus 49.4 ± 16.3, p < 0.001). There were no significant differences between respondents’ gender (p = 0.427) and educational level (p = 0.376) compared with the non-responders. The results in this study are presented for 445 persons (51.7%) with an increased weight-related health risk.

### Demographics and beliefs about weight and health

The participants in this study with an increased WRHR were on average 56.4 years old and the vast majority were native Dutch (96%). A large proportion had an advanced level of education (60.1%) and the majority were married (71.3%). The largest group had a mild (39.3%) or moderately (45.8%) increased WRHR. A majority did not exercise for 30 minutes at a moderate-level on at least five days a week (59.1%). Furthermore, about one out of two persons had an accurate perception of their weight (50.5%) or perceived their general health as good (55.7%).

### Perceptions and expectations regarding care providers giving dietary advice

**Figure 3:**
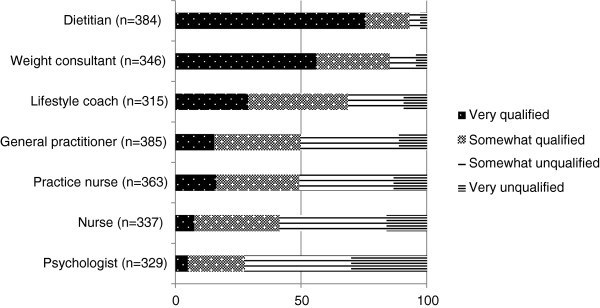
Perception of care providers’ suitability to give dietary advice, among persons with an increased weight-related health risk (%).

**Figure 4:**
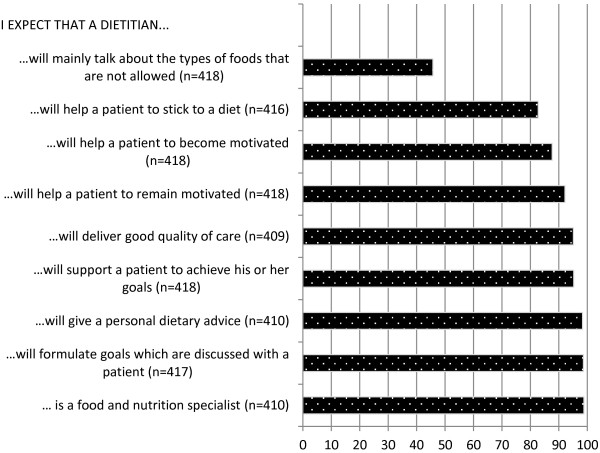
Expectations of dietitians, among persons with an increased weight-related health risk who (fully) agreed with the statements (%).

The majority of persons with an increased WRHR believed that dietitians were most qualified to give dietary advice, followed by weight consultants and lifestyle coaches. General practitioners and practice nurses shared fourth place (see Figure 3). Many respondents did not have an opinion about the role of care providers in giving dietary advice, which was apparent from the relatively high number of blanks per item. The expectations that respondents had of dietitians are described in Figure 4. The average 4-point scale expectations-score (mean ± sd) was 3.5 ± 0.5, meaning that respondents generally had positive expectations of dietitians. Respondents who were not aware of what a dietitian does (n = 17) and those with up to four missing items on expectations (n = 7) were not included in the average scale score on expectations. Three additional statements were presented in order to compare the results regarding expectations of dietitians to expectations of other care providers or diet methods. The majority of respondents believed or fully believed that dietitians are better than other care providers or diet methods, since they: deliver better quality of care (83.1%), give individual dietary advice (96.4%), or help patients to remain motivated (90.9%). Persons with an increased WRHR reported a trust-rating in dietitians of 7.3 ± 1.2 on a scale from 1–10, where 82.9% reported a 7 or higher.

### What type of persons were ready to lose weight?

Overall, 55.1% (n = 245) of persons with an increased WRHR were ready to lose weight (see Figure 2). Table 1 shows the unadjusted relationship between predisposing factors and reported readiness to lose weight, stratified by WRHR. Results of multivariate regression analyses varied between levels of WRHR (see Table 2). Respondents with a mildly increased WRHR had significantly higher odds for readiness to lose weight in case they perceived the dietitian as suitable caregiver, or in case they had an accurate perception of weight. Subsequently, respondents with a moderately increased WRHR had significantly higher odds for readiness to lose weight in individuals with an accurate perception of weight, in those with an advanced or high educational level and in those with higher expectation scores of dietitians. Furthermore, individuals with a severely or very severely increased WRHR and not married had a higher odds for readiness to lose weight compared to married individuals.

**Table 1:** Influence of determinants on reported readiness to lose weight, stratified by weight related health risk*

**Characteristic**	**Mild WRHR**		**Moderate WRHR**		**Severe WRHR**	
	**N = 175**		**N = 204**		**N = 66**	
	**Ready**	**Not ready**	**Ready**	**Not ready**	**Ready**	**Not ready**
	**N = 102**	**N = 73**	**N = 101**	**N = 103**	**N = 42**	**N = 24**
Age category, n (%)	P = 0.121		**P = 0.044**		P = 0.559	
20 – 39.9	28(73.7)	10(26.3)	21(67.7)	10(32.3)	5(55.6)	4(44.4)
40 – 49.9	17(51.5)	16(48.5)	13(59.1)	9(40.9)	5(55.6)	4(44.4)
50 – 59.9	26(60.5)	17(39.5)	23(52.3)	21(47.7)	9(81.8)	2(18.2)
60+	31(50.8)	30(49.2)	44(41.1)	63(58.9)	23(62.2)	14(37.8)
Gender, n (%)	**P = 0.002**		**P = 0.003**		P = 0.670	
Male	47(48.0)	51(52.0)	42(39.6)	64(60.4)	18(66.7)	9(33.3)
Female	55(71.4)	22(28.6	59(60.2)	39(39.8)	24(61.5)	15(38.5)
Ethnic background, n (%)	P = 0.197		P = 0.649		P = 0.548	
Western	8(80.0)	2(20.0)	3(50.0)	3(50.0)	1(33.3)	2(66.7)
Non-Western	94(57.0)	71(43.0)	98(49.5)	100(50.5)	41(65.1)	22(34.9)
Educational level, n (%)	P = 0.996		**P = 0.002**		P = 0.754	
Low (primary, lower vocational)	7(58.3)	5(41.7)	6(20.0)	24(80.0)	6(60.0)	4(40.0)
Advanced (secondary, pre-university)	58(58.6)	41(41.4)	61(50.8)	59(49.2)	26(66.7)	13(33.3)
High (bachelor’s degree or more)	33(57.9)	24(42.1)	29(60.4)	19(39.6)	8(57.1)	6(42.9)
Marital status, n (%)	P = 0.491		P = 0.234		**P = 0.039**	
Married	75(55.6)	60(44.4)	68(47.6)	75(52.5)	24(63.2)	14(36.8)
Divorced	7(63.6)	4(36.4)	9(45.0)	11(55.0)	8(88.9)	1(11.1)
Widowed	3(60.0)	2(40.0)	8(44.4)	10(55.6)	6(85.7)	1(14.3)
Never married	16(72.7)	6(27.3)	16(69.6)	7(30.4)	4(33.3)	8(66.7)
Net monthly household income, n (%)	P = 0.484		**P = 0.005**		P = 0.978	
Up to €1450	11(61.1)	7(38.9)	11(39.3)	17(60.7)	10(62.5)	6(37.5)
€1450 < €2100	21(50.0)	21(50.0)	28(44.4)	35(55.6)	7(58.3)	5(41.7)
€2100 < €2900	28(54.9)	23(45.1)	24(40.7)	35(59.3)	13(61.9)	8(38.1)
€2900 +	40(64.5)	22(35.5)	38(70.4)	16(29.6)	10(66.7)	5(33.3)
Physical activity, n (%)	**P = 0.046**		P = 0.864		P = 0.587	
< 5 days/week	63(65.0)	34(35.0)	61(50.0)	61(50.0)	29(65.9)	15(34.1)
≥ 5 days/week	39(50.0)	39(50.0)	40(48.8)	42(51.2)	13(59.1)	9(40.9)
Accurate perception of weight, n (%)	**P < 0.001**		**P < 0.001**		P = 0.615	
No	40(38.5)	64(61.5)	36(33.6)	71(66.4)	5(62.5)	3(37.5)
Yes	61(88.4)	8(11.6)	65(67.7)	31(32.3)	37(63.8)	21(36.2)
Self-perceived general health, n (%)	P = 0.474		P = 0.536		P = 0.532	
Poor/Fair	5(50.0)	5(50.0)	18(50.0)	18(50.0)	15(71.4)	6(28.6)
Good	51(63.0)	30(37.0)	65(51.2)	62(48.8)	22(62.9)	13(37.1)
Very good/excellent	43(54.4)	36(45.6)	16(41.0)	23(59.0)	4(50.0)	4(50.0)
Perceive dietitian as suitable caregiver, n (%)	P = 0.082		P = 0.291		P = 0.461	
No	8(88.9)	1(11.1)	6(75.0)	2(25.0)	4(44.4)	5(55.6)
Yes	82(56.9)	62(43.1)	88(53.0)	78(47.0)	30(62.5)	18(37.5)
Expectations of dietitian score	P = 0.186		**P = 0.007**		P = 0.189	
mean ± sd	3.5 ± 0.5	3.6 ± 0.5	3.6 ± 0.4	3.4 ± 0.5	3.6 ± 0.4	3.4 ± 0.5
Trust-rating in dietitians	P = 0.489		P = 0.197		P = 0.941	
mean ± sd	7.5 ± 0.9	7.3 ± 1.3	7.4 ± 1.0	7.2 ± 1.1	6.9 ± 1.9	7.0 ± 1.3

*Unadjusted results from bivariate analysis.

**Table 2:** Factors associated with reported readiness to lose weight, stratified by weight related health risk - results from multivariate logistic regression analysis

	**Odds ratio**	***P-value***	**(95% CI)**
**Final model: ready to lose weight, mild WRHR**			
Accurate perception of weight			
No (reference)			
Yes	14.16	<0.001	(5.71; 35.07)
Perceive dietitian as suitable caregiver			
No (reference)			
Yes	0.09	0.025	(0.01; 0.74)
**Final model: ready to lose weight, moderate WRHR**			
Educational level			
Low (reference)			
Advanced	4.83	0.006	(1.58; 14.78)
High	7.49	0.001	(2.19; 25.63)
Accurate perception of weight			
No (reference)			
Yes	3.68	<0.001	(1.91; 7.10)
Expectations of dietitian score	2.70	0.011	(1.26; 5.80)
**Final model: ready to lose weight, severe WRHR**			
Marital status			
Married (reference)			
Divorced	4.67	0.167	(0.53; 41.3)
Widowed	3.50	0.268	(0.38; 32.1)
Never married	0.29	0.078	(0.07; 1.14)

### What are the most important reasons for not being ready to lose weight?

**Figure 5:**
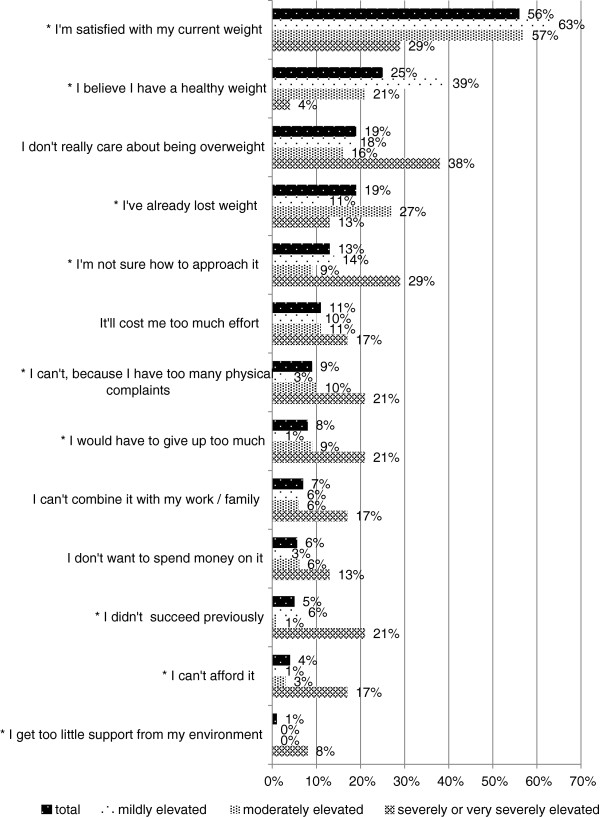
**Reasons for not being ready to lose weight by weight-related health risk (% of n = 200).** *There was a statistically significant difference (p < 0.05) between the three levels of WRHR.

About half of the respondents with increased WRHR were not ready to lose weight (n = 200). The main reasons given varied according to level of WRHR (see Figure 5). Those with a mildly increased WRHR were more often satisfied with their current weight or believed they were at a healthy weight compared to those with a higher level of WRHR. Persons with a severely or very severely increased WRHR were more often not ready to lose weight compared to persons with a lower level of WRHR because they: were not sure how to approach weight loss, had too many physical complaints, would have to give up too much, did not succeed previously, received less support from family, or could not afford it.

### What type of persons intended to use weight-related care?

The largest group of respondents with an increased weight-related health risk who were ready to lose weight preferred individual weight loss methods without help from others, for example by starting to eat healthier and exercise more often (82.0% of n = 245). Eleven per cent (n = 26 of 245) intended to use weight-related care from a care provider, especially from dietitians (n = 12).

Having the intention to use weight-related care was significantly higher for those with a moderately, severely, or very severely elevated level of WRHR compared to those with a mild WRHR. In addition, those who perceived their general health as poor more often intend to use weight-related care (see Table 3).

**Table 3:** Influence of determinants on reported intention to use weight-related care*

	**Intention to use weight-related care from a care provider**	
**Characteristic**	**Yes**	**No**
	**N = 26**	**N = 219**
Age category, n (%)	P = 0.090	
20 – 39.9	4(7.4)	50(92.6)
40 – 49.9	5(14.3)	30(85.7)
50 – 59.9	2(3.5)	56(96.6)
60+	15(15.3)	83(84.7)
Gender, n (%)	P = 0.491	
Male	13(12.2)	94(87.9)
Female	13(9.4)	125(90.6)
Ethnic background, n (%)	P = 0.622	
Western	26(11.2)	207(88.8)
Non-Western	0(0.0)	12(100)
Educational level, n (%)	P = 0.208	
Low (primary, lower vocational)	4(21.1)	15(78.9)
Advanced (secondary, pre university)	15(10.3)	130(89.7)
High (bachelor’s degree or more)	5(7.1)	65(92.9)
Marital status, n (%)	P = 0.226	
Married	14(8.4)	153(91.6)
Divorced	3(12.5)	21(87.5)
Widowed	4(23.5)	13(76.5)
Never married	5(13.9)	31(86.1)
Net monthly household income, n (%)	P = 0.920	
Up to €1450	3(9.4)	29(90.6)
€1450 < €2100	7(12.5)	49(87.5)
€2100 < €2900	7(10.8)	58(89.2)
€2900 +	8(9.1)	80(90.9)
Physical activity, n (%)	P = 0.596	
< 5 days/week	15(9.8)	138(90.2)
≥ 5 days/week	11(12.0)	81(88.0)
Accurate perception of weight, n (%)	P = 0.560	
No	7(8.6)	74(91.4)
Yes	18(11.0)	145(89.0)
Self-perceived general health, n (%)	P = **0.016**	
Poor/Fair	8(21.1)	30(78.9)
Good	15(10.9)	123(89.1)
Very good/excellent	2(3.2)	61(96.8)
Weight-related Health Risk:	P **< 0.001**	
Mild	2(2.0)	100(98.0)
Moderate	11(10.9)	90(89.1)
Severe or very severe	13(31.0)	29(69.1)
Perceive dietitian as suitable caregiver, n (%)	P = 0.440	
No	1(5.6)	17(94.4)
Yes	23(11.5)	177(88.5)
Expectations of dietitian score	P = 0.404	
mean ± sd	3.5 ± 0.4	3.5 ± 0.4
Trust-rating in dietitians	P = 0.804	
mean ± sd	7.3 ± 1.7	7.4 ± 1.1

^*^Unadjusted results from bivariate analysis. The data involves persons with an increased weight related health risk and reported readiness to lose weight.

Most of the those with the intention to use weight-related care reported to have received dietary advice from a care provider in the past (n = 23 of 26). Overall, 33.9% of the persons who were ready to lose weight (12.8% mild WRHR, 46.0% moderate WRHR, 57.5% severe WRHR) reported to have received dietary advice from a care provider in the past (results not in table).

## Discussion

The current study provides insight into readiness to lose weight, intention to use weight-related care, and influencing factors, in an overweight population with weight-related health risks. This information is important for the development of strategies for successful obesity prevention and management.

Results show that 52% of the study sample had an elevated level of weight-related health risk, and were therefore in medical need of obesity prevention or management. Perceived need for obesity prevention and management was considerably lower since about half of them were ready to lose weight, i.e. they were in the preparation, active, or maintenance stage for weight loss. These results are comparable with results from a survey conducted among primary care patients [13]. Only eleven per cent of those who were planning to lose weight preferred to do so with help from a care provider and one in three reported to have received dietary advice from a care provider in the past. The extent to which the dietary advice helped them is unknown; however, most were not planning to lose weight with help from a health provider again. Future research in evaluating patient experiences with dietary treatment is therefore recommended. From the results of this study, it is not clear why there is an overall low intention to use weight-related care. Weight-related care seeking might possibly be higher if more effective strategies for the prevention of overweight and obesity were available at population level. The lack of reimbursement for dietary treatment in some individuals with a mildly or moderately elevated level of WRHR would most likely not have influenced their intention to use weight-related care from a care provider. Most people believed that dietary treatment was reimbursed and this did not vary between WRHR groups, nor was it significantly associated with the intention to use weight-related care (results not shown).

The discrepancy between perceived need for and intended use of weight-related care can be explained by the relatively large group of people who were ready to lose weight but preferred to do so individually. However, individual weight loss attempts often prove to be less effective than weight management programmes [21]. Obesity prevention initiatives should therefore include the advice that weight loss without skilled supervision usually does not lead to successful weight loss and may do more harm than good. In addition, monitoring of weight change and weight loss plans should be encouraged [22]. If overweight patients fail to lose weight on their own, care providers could refer them for obesity management. Care providers may in turn offer evidence-based effective lifestyle advice with realistic levels of effort and outcomes (5-10% weight loss is associated with meaningful improvements in health related risk factors [23,24]). In addition, they may emphasise the importance of weight relapse prevention and use techniques such as motivational interviewing and elements of self-determination theory (such as autonomy, competence, and relatedness) that have been shown to predict long-term success in weight management [25].

Even though there was a large group that was willing to lose weight, there remained a sizable group of overweight and obese people who need encouragement to start losing weight. This group consisted mainly of individuals who were about 60 years of age, male, with a low level of education, a net monthly household income between €1450 < €2100, an inaccurate perception of their own weight, and a moderately elevated level of WHRH. Most of those who were not ready to lose weight were precontemplators, since they seemed uninformed about the consequences of their weight (e.g. were satisfied with their current weight, believed they had a healthy weight or did not seem to care about being overweight), or had previously tried to change but became demoralised about their ability to do so [9]. Therefore, obesity prevention initiatives should attempt to focus on increasing awareness of the seriousness of their condition and on offering individually appropriate weight management programmes. General practitioners can play an important role in stimulating behaviour change regarding weight loss. Some studies have shown that general practitioners discuss weight with less than half of obese patients who visit their practice [22,26]. More discussions about weight management or referral options might help patients become more willing to engage in weight-behaviour change and receive weight-related care.

Although dietitians are not the only professionals qualified to give dietary advice, the majority of respondents believed that dietitians were the most qualified care providers in the area of dietary advice and they had generally positive expectations of dietitians. The level of trust (83%) in dietitians was high compared with Dutch public trust ratings in complementary and alternative medicine (45%) and was comparable to public trust ratings in general practitioners (89%) and physical therapists (87%) [27]. Furthermore, respondents believed that psychologists were the least suitable to give dietary advice. The role of psychologists in weight management, as described in clinical guidelines, is mainly focussed on providing psychological support for behaviour change (4). The psychological component of weight management might be quit unknown amongst the population.

Further results show several predisposing factors associated with readiness to lose weight. Depending on one’s WRHR, higher odds for readiness to lose weight were observed for those who perceive the dietitian was a suitable caregiver and those with higher expectations of dietitians. Therefore, promoting dietitians’ activities may potentially stimulate the motivation to change weight, which can be seen as a prerequisite for obesity management. In addition, persons with a moderately increased WRHR and higher educational level were associated with being at advanced stages of readiness for weight loss. A survey of the U.S. population also reported that sociodemographics were associated with trying to lose weight [10]. One of the underlying explanations for differences in socio economic status on readiness to change may be related to beliefs and lack of knowledge about health risks, e.g. people with a low socioeconomic status might not see the health risks of being overweight [28]. Furthermore, sociologists argue about the importance of marital status in affecting adults’ body weight. Results from our study showed that divorce, widowhood and never being married was significantly associated with being ready to lose weight in individuals with severe WRHR, compared to those who are married. This result was in line with a systematic literature review reporting that transitions into marriage were associated with weight gain, whereas transitions out of marriage (through divorce and widowhood) were associated with triggering weight loss [29]. Further results showed that accurately perceiving oneself as being overweight or obese is considered to be an important aspect of weight change, which was in agreement with others [30]. Overall, the results on readiness to lose weight need to be confirmed by others, as the observed associations are inconsistent among different levels of WRHR.

Regarding intention to use weight-related care, the results show that persons who perceived their general health as poor more often have this intention. Additionally, adults with a risk factor for cardiovascular diseases, co-morbidities and/or obesity were more inclined to seek weight-related care than overweight adults without risk factors for CVD. Accordingly, these findings indicate that the type of individuals seeking weight-related care from a care provider match the guidelines for obesity management. Multivariate regression analysis stratified by weight related health risk was not applied, considering the small sample size of persons intending to use weight-related care from a care provider. Consequently, these results need to be confirmed within a larger sample. A strength of this study was the representative sample of Dutch adults, who regularly receive online health care surveys. Since the panel members were familiar with online surveys, we do not expect this would have biased the response. An important limitation of our study is the lack of generalisability of the results. Our study population consisted mainly of relatively older people, and thus the results may be less representative of younger age groups. Moreover, the response rate was relatively low compared with the response rate of more than 70% usually obtained from this panel [17]. The topic of the questionnaire may not have been of interest to all. This could potentially have influenced the prevalence of people with an elevated level of WRHR. However, the prevalence of overweight and obesity is comparable to national estimates of self-reported data [2], as well as national estimates of prevalence by gender and age group [31]. Additionally, potential sources of response bias may exist, as the questions on lifestyle and health were self-reported. Evidence suggests that women often under-report their weight and men often over-report height [32]. This may have resulted in under-classification of WRHR groups. However, since the prevalence of overweight and obesity is comparable to national estimates of self-reported data, we do not expect weight to be very much under-reported. Furthermore, the results in this study are likely to be overestimated because people tend to be optimistic about their behaviour and intentions. Nevertheless, self-report is the only means of capturing patients’ stage of behaviour change.

## Conclusion

The medical need for obesity prevention and management is high; however, about half of the Dutch adults who are in need of weight-related care are ready to lose weight. Most have the intention to lose weight individually, and only a few have the intention to use weight-related care.

Dietitians were perceived to be the most qualified health professionals to give dietary advice. Weight-related care seeking was not influenced by perceptions, expectations or trust in dietitians. In general, weight-related care seeking was higher for adults who perceived their health as poor. In addition, they more often have a risk factor for cardiovascular diseases, co-morbidities and/or obesity compared with overweight adults without risk factors for cardiovascular diseases, which matches the guidelines for obesity. For the group of individuals who are ready to lose weight, obesity prevention initiatives should focus on monitoring weight change and providing weight loss plans and timely referrals for obesity management. Moreover, many people are not ready to lose weight. For this group, strategies for behaviour change may depend on weight related health risk, perceptions of weight and dietitians, educational level and marital status. Obesity prevention initiatives should focus on increasing their awareness of the seriousness of their condition and offer individually appropriate weight management programmes.

## Competing interests

The authors declare that they have no competing interests.

## Authors’ contributions

All authors were involved in the conception and design of the study and in the acquisition and interpretation of data. JT performed the statistical analyses and drafted the paper. All authors have critically reviewed the manuscript and have approved the final version submitted for publication.

## Pre-publication history

The pre-publication history for this paper can be accessed here:

http://www.biomedcentral.com/1471-2458/14/582/prepub

## Supplementary Material

Additional file 1 Questionnaire about your lifestyle and your opinion of dietary treatment. Click here for file
