# Supplementary material for: Overweight and obese adults have low intentions of seeking weight-related care: a cross-sectional survey
Source: BMC Public Health. 2014 Jun 11;14:582. doi: 10.1186/1471-2458-14-582 (PMC4085466; doi:10.1186/1471-2458-14-582)
Supplement: Additional file 1 — Questionnaire about your lifestyle and your opinion of dietary treatment. [file 1471-2458-14-582-S1.pdf]

Additional file 1. Questionnaire about your lifestyle and your opinion of dietary treatment

---

A. Questions about your background

---

1. What is your date of birth?

   (day – month – year)

2. Are you a man or a woman?

   - ❑ Man
   - ❑ Woman

B. Questions about your lifestyle

Physical activity

3. How many days a week do you exercise for at least 30 minutes per day? This may be spread over the day, in units of 10 minutes. Exercise is defined as any physical activity which raises your heart rate and increases your breathing.

   - ❑ 0 days a week
   - ❑ 1 day a week
   - ❑ 2 days a week
   - ❑ 3 days a week
   - ❑ 4 days a week
   - ❑ 5 days a week
   - ❑ 6 days a week
   - ❑ 7 days a week

Weight

4. What is your current height?

__ __ __ centimetres

5. What is your current weight? If you are pregnant, please report your pre-pregnancy weight.

__ __ __ kilograms

6. To what extent do you agree with the following statement: I believe I am too heavy.

- ❑ Strongly disagree
- ❑ Somewhat disagree
- ❑ Somewhat agree
- ❑ Strongly agree

7. Do you plan to start losing weight?

- ❑ No
- ❑ Yes, I'm planning to change but not in the short term
- ❑ Yes, I'm planning to change during the next month
- ❑ Yes, I am currently changing

8. What are the most important reasons you're not planning to lose weight, or at least not in the short-term? We would like to ask you to fill out three reasons maximum.

- ❑ I would like to gain weight
- ❑ I believe I have a healthy weight
- ❑ I'm satisfied with my current weight
- ❑ I've already lost weight

- ❑ I don't really care about being overweight
- ❑ I'm not sure how to approach it
- ❑ I can't, because I have too many physical complaints
- ❑ It'll cost me too much effort
- ❑ I would have to give up too much
- ❑ I didn’t succeed previously
- ❑ I get too little support from my environment (such as support from family/friends)
- ❑ I can’t combine it with my work/family commitments
- ❑ I can't afford it
- ❑ I don't want to spend money on it
- ❑ Other reason, namely…

→ *Continue to part C, unless “Yes,…” was reported at question 7*

Weight loss method

9. You have previously mentioned having plans to start losing weight. What are your weight loss plans? Multiple responses are allowed.

- ❑ Individual (healthier diet, more exercise)
- ❑ Diet from a book, magazine from the internet
- ❑ Meal replacements
- ❑ Diet pills
- ❑ Surgery (for example gastric bypass surgery)
- ❑ Support via internet (for example websites, chat, App)
- ❑ Support from courses (for example weight watchers)
- ❑ Support from a care giver (for example general practitioner, dietitian) → *Continue to questions 10 and 11.*
- ❑ I don’t know (yet)

→ *Continue to part C, unless “Support from a care provider” was filled-out at question 9*

10. How would you like to receive weight loss support from a care provider? If you currently receive support from a care provider, what method is applied? Multiple responses are allowed.

- ❑ Group sessions
- ❑ One on one consultations (individual support)
- ❑ E-mail, chat or text
- ❑ Phone
- ❑ Other method, namely…
- ❑ I don’t know (yet)

11. From which care provider would you like to receive weight-loss support?

- ❑ General practitioner
- ❑ Practice nurse
- ❑ Dietitian
- ❑ Weight consultant
- ❑ Lifestyle coach
- ❑ Nurse
- ❑ Psychologist
- ❑ Multiple care providers
- ❑ Other care provider, namely: ....
- ❑ I don’t know (yet)

C. Your opinion of dietary treatment

A diet consists of rules on what foods you may or may not eat. Many diets exist. A diet can be followed independently or with help from a care provider. For many individuals, diets are radical and difficult to maintain. During dietary treatment, clients will be encouraged to continue with the diet.

12. Have you ever received dietary treatment from a care provider?

- ❑ No
- ❑ Yes
- ❑ I don't know

13. Please indicate the extent to which you rate the following care providers as qualified to give dietary advice?

	Very unqualified	*Somewhat unqualified*	*Somewhat qualified*	*Very qualified*	*I don't know*
a. General practitioner	❑	❑	❑	❑	❑
b. Practice nurse	❑	❑	❑	❑	❑
c. Dietitian	❑	❑	❑	❑	❑
d. Weight consultant	❑	❑	❑	❑	❑
e. Lifestyle coach	❑	❑	❑	❑	❑
f. Nurse	❑	❑	❑	❑	❑
g. Psychologist	❑	❑	❑	❑	❑

14. Do you know what a dietitian does?

- ❑ Not at all → *Continue to question 18*
- ❑ Slightly
- ❑ Largely
- ❑ Fully

15. We would like to know about your expectations of dietitians. Please indicate the extent to which you agree with the following expectations of dietitians.

I expect that a dietitian …	*Totally disagree*	*Slightly*	*Largely*	*Fully agree*
a. …is a food and nutrition specialist	❑	❑	❑	❑
b. …will deliver good quality of care	❑	❑	❑	❑
c. …will **mainly** talk about the types of foods that are no longer allowed	❑	❑	❑	❑
d. …will give personal dietary advice	❑	❑	❑	❑
e. …will formulate goals which are discussed with the patient	❑	❑	❑	❑
f. … will support a patient to achieve his or her goals	❑	❑	❑	❑
g. …will help a patient **to become** motivated	❑	❑	❑	❑
h. …will help a patient **to remain** motivated	❑	❑	❑	❑
i. … will help a patient to stick to a diet	❑	❑	❑	❑

16. Please indicate the extent to which you agree that dietitians are better in comparison with other care providers or diet methods?

The dietitian is better since he or she…	*Totally disagree*	*Slightly*	*Largely*	*Fully agree*	*I don't know*
a. … delivers better quality of care	❑	❑	❑	❑	❑
b. … gives individual dietary advice	❑	❑	❑	❑	❑
c. … helps a patient to remain motivated	❑	❑	❑	❑	❑

17. Please give a report mark between 1 and 10 for your trust in dietitians?

1	2	3	4	5	6	7	8	9	10
❑	❑	❑	❑	❑	❑	❑	❑	❑	❑

18. Please indicate whether you have had any of the following diseases, health conditions or complaints in the past 12 months? Multiple responses are allowed.

- ❑ I did not have any diseases or health conditions
- ❑ Diabetes type 2
- ❑ Heart diseases/conditions
- ❑ High blood pressure
- ❑ High blood sugar
- ❑ Diseases of the joints
